# A 63-year-old woman presenting with a synovial sarcoma of the hand: a case report

**DOI:** 10.1186/1752-1947-6-385

**Published:** 2012-11-13

**Authors:** Diogo Casal, Ana Isabel Ribeiro, Manuela Mafra, Conceição Azeda, Carlos Mavioso, Maria Manuel Mendes, Maria Manuel Mouzinho

**Affiliations:** 1 Plastic and Reconstructive Surgery Resident, São José Hospital, Lisbon, Portugal; 2 Pathology Resident, São José Hospital, Lisbon, Portugal; 3 Pathology Senior Consultant, São José Hospital, Lisbon, Portugal; 4 Plastic and Reconstructive Surgery Senior Consultant, São José Hospital, Lisbon, Portugal; 5 Head of the Hand Surgery Clinic, São José Hospital, Lisbon, Portugal; 6 Plastic and Reconstructive Surgery Department, Hospital de São José, Rua José António Serrano, 1150-199, Lisbon, Portugal

**Keywords:** Synovial sarcoma, Hand, Surgery, Malignant tumor

## Abstract

**Introduction:**

Synovial sarcoma is a high-grade, soft-tissue sarcoma that most frequently is located in the vicinity of joints, tendons or bursae, although it can also be found in extra-articular locations. Most patients with synovial sarcoma of the hand are young and have a poor prognosis, as these tumors are locally aggressive and are associated with a relatively high metastasis rate. According to the literature, local recurrence and/or metastatic disease is found in nearly 80% of patients. Current therapy comprises surgery, systemic and limb perfusion chemotherapy, and radiotherapy. However, the 5-year survival rate is estimated to be only around 27% to 55%. Moreover, most authors agree that synovial sarcoma is one of the most commonly misdiagnosed malignancies of soft tissues because of their slow growing pattern, benign radiographic appearance, ability to change size, and the fact that they may elicit pain similar to that caused by common trauma.

**Case presentation:**

We describe an unusual case of a large synovial sarcoma of the hand in a 63-year-old Caucasian woman followed for 12 years by a multidisciplinary team. In addition, a literature review of the most pertinent aspects of the epidemiology, diagnosis, treatment and prognosis of these patients is presented.

**Conclusion:**

Awareness of this rare tumor by anyone dealing with hand pathology can hasten diagnosis, and this, in turn, can potentially increase survival. Therefore, a high index of suspicion for this disease should be kept in mind, particularly when evaluating young people, as they are the most commonly affected group.

## Introduction

Synovial sarcoma of the hand is an extremely rare entity that carries a worse prognosis than that of most soft-tissue sarcomas [1,2]. It has been estimated that, on average, even hand surgeons will encounter only one or two undiagnosed soft-tissue sarcomas of the upper extremity during the entire duration of their careers [1].

In addition, the majority of authors agree that synovial sarcoma is one of the most commonly misdiagnosed malignancies of the soft tissues, owing to its slow growing pattern, benign radiographic appearance, ability to change size, and their often eliciting pain similar to that caused by common trauma [3,4]. Hence, synovial sarcoma patients are often diagnosed initially as having myositis, hematoma, synovitis, tendonitis, bursitis or other common disorders [3,4].

To make matters worse, primary synovial sarcoma size and initial status at presentation have been shown to strongly affect survival [3,5]. Therefore, a high index of suspicion for this disease should be kept in mind, particularly when evaluating young people, as they are the most commonly affected group [3,4]. Herein we describe the clinical case of a large synovial sarcoma of the hand in a 63-year-old woman followed up for 12 years. Moreover, a brief review of this unusual sarcoma is presented.

## Case presentation

**Figure 1:**
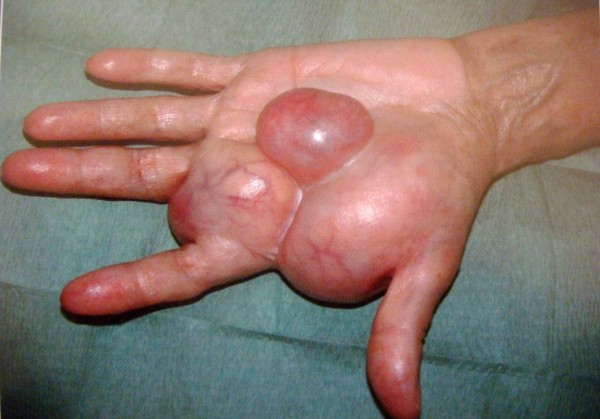
Palmar aspect of the left hand showing a large nodular mass over the first four rays.

**Figure 2:**
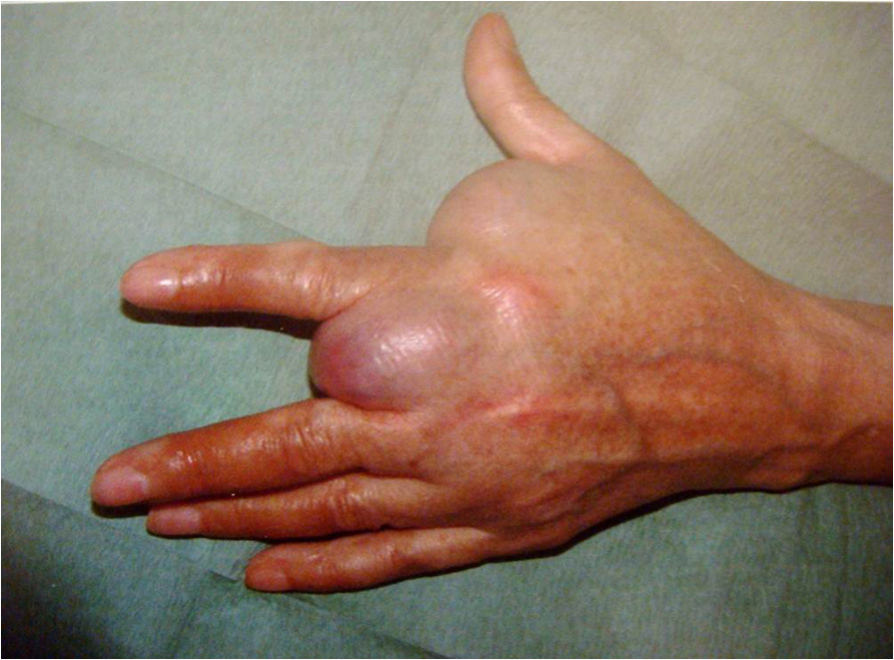
Dorsal aspect of the hand with the protruding mass in the first and second interdigital web spaces preventing complete finger adduction.

**Figure 3:**
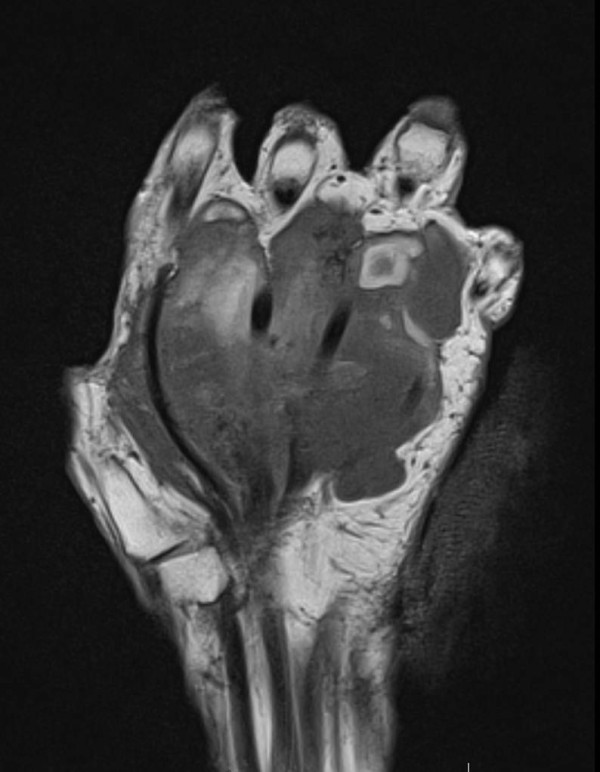
Magnetic resonance image of a coronal section of the hand showing a large mass around the palmar structures of the hand, but with no bone involvement.

**Figure 4:**
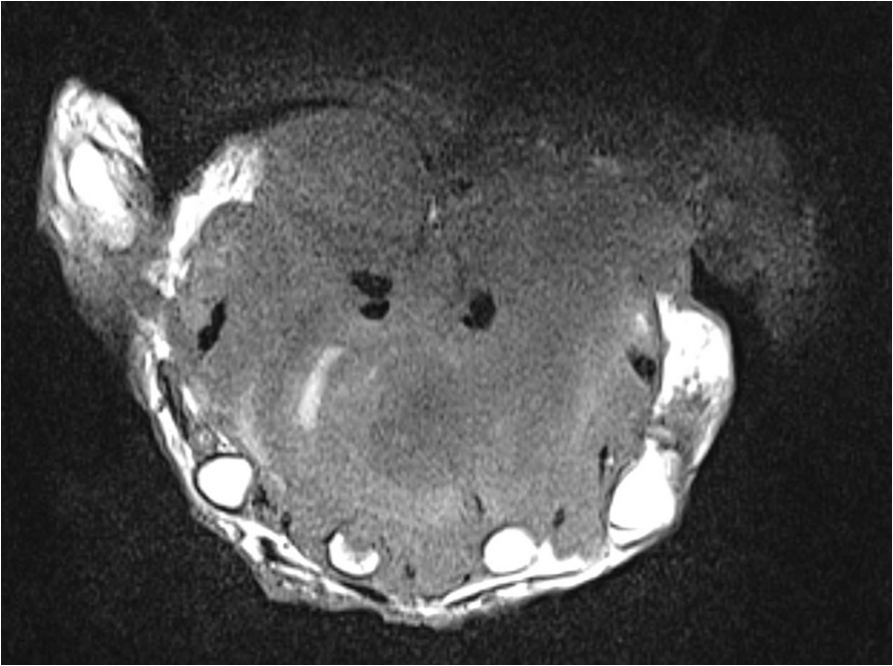
Magnetic resonance image of a transverse section of the hand showing a large mass around the palmar structures of the hand, but with no bone involvement.

**Figure 5:**
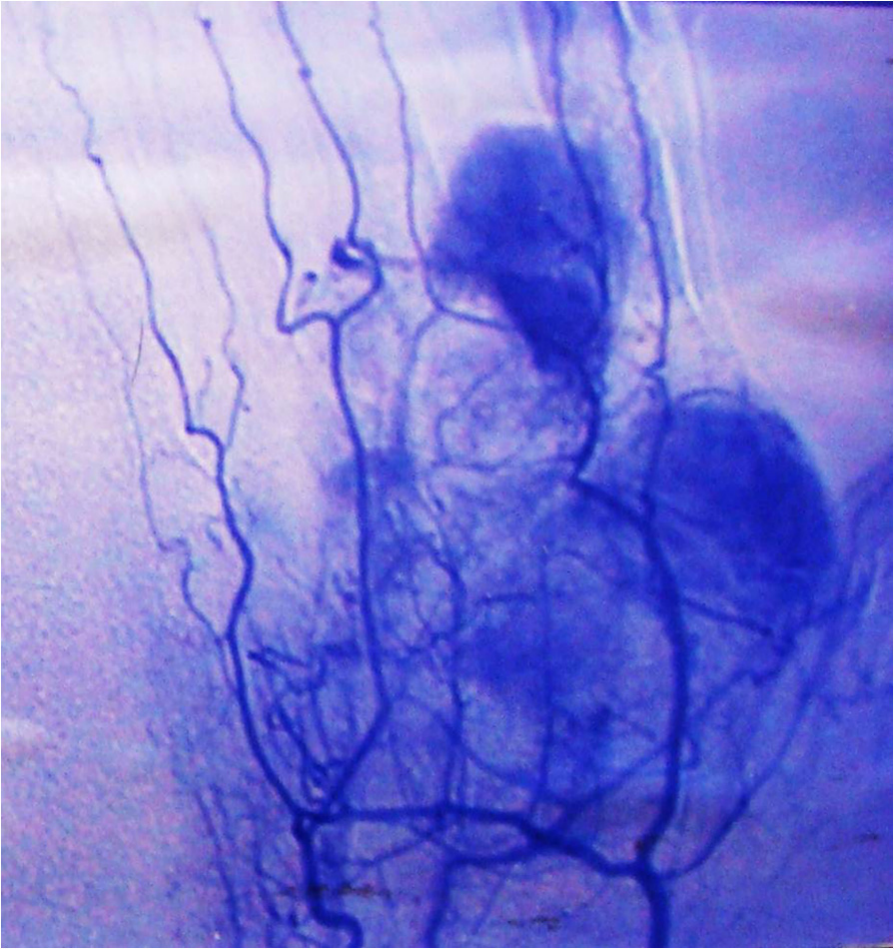
Pre-operative angiogram showing the rich blood supply to the tumor, arising from the deep palmar arch, from the external branch of the superficial palmar arch and from the palmar digital arteries of the first, second and third fingers.

A 63-year-old, right-handed Caucasian woman with an unremarkable medical history was referred to the hand clinic at our institution with a large firm mass in her left hand that had been growing steadily over the previous 3 years (Figures 1 and 2). The mass had grown to a point that it interfered with many of her daily life activities. She complained of pain and occasional paresthesia in the first three rays of the hand. Ultrasound examination was inconclusive. Magnetic resonance imaging (MRI) was performed, which revealed a large mass spanning almost the entire palmar aspect of the hand, superficially to the flexor tendons and with no evidence of bone involvement (Figures 3 and 4). A pre-operative angiogram showed a tumor with a rich blood supply (Figure 5). The tumor vessels were given off by the deep palmar arch, the external branch of the superficial palmar arch and the palmar digital arteries of the first, second and third fingers. With the patient under local anesthesia, an incisional biopsy was performed, which identified a synovial sarcoma.

**Figure 6:**
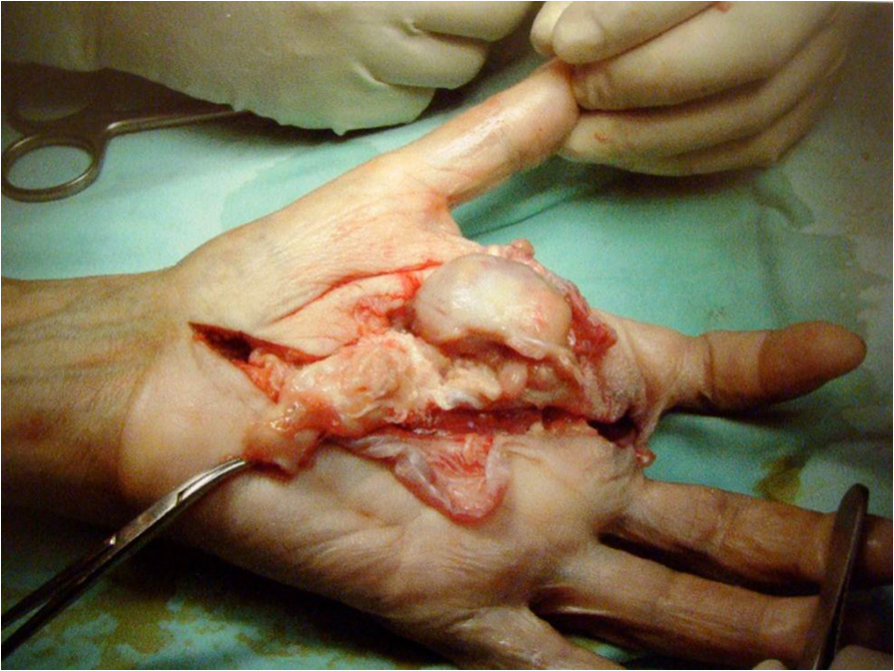
Intraoperative view of the excised specimen.

**Figure 7:**
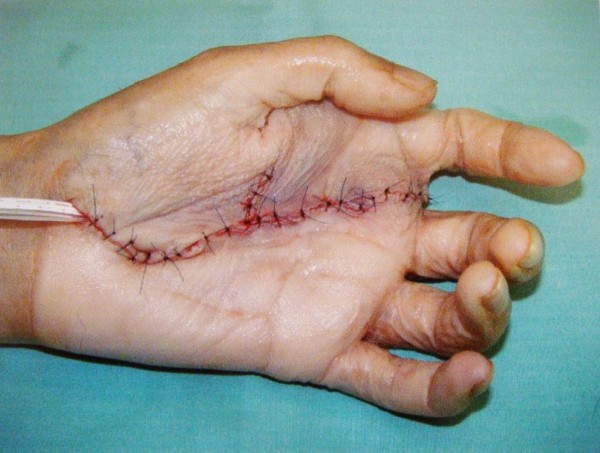
At the end of the surgery, all digits were spared.

**Figure 8:**
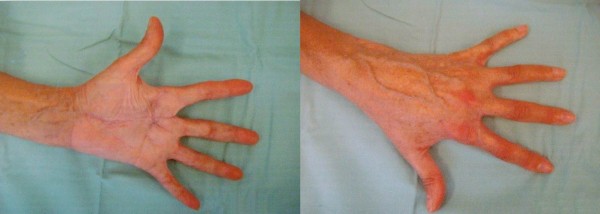
One month post-operatively, the patient had a good functional and aesthetic result.

**Figure 9:**
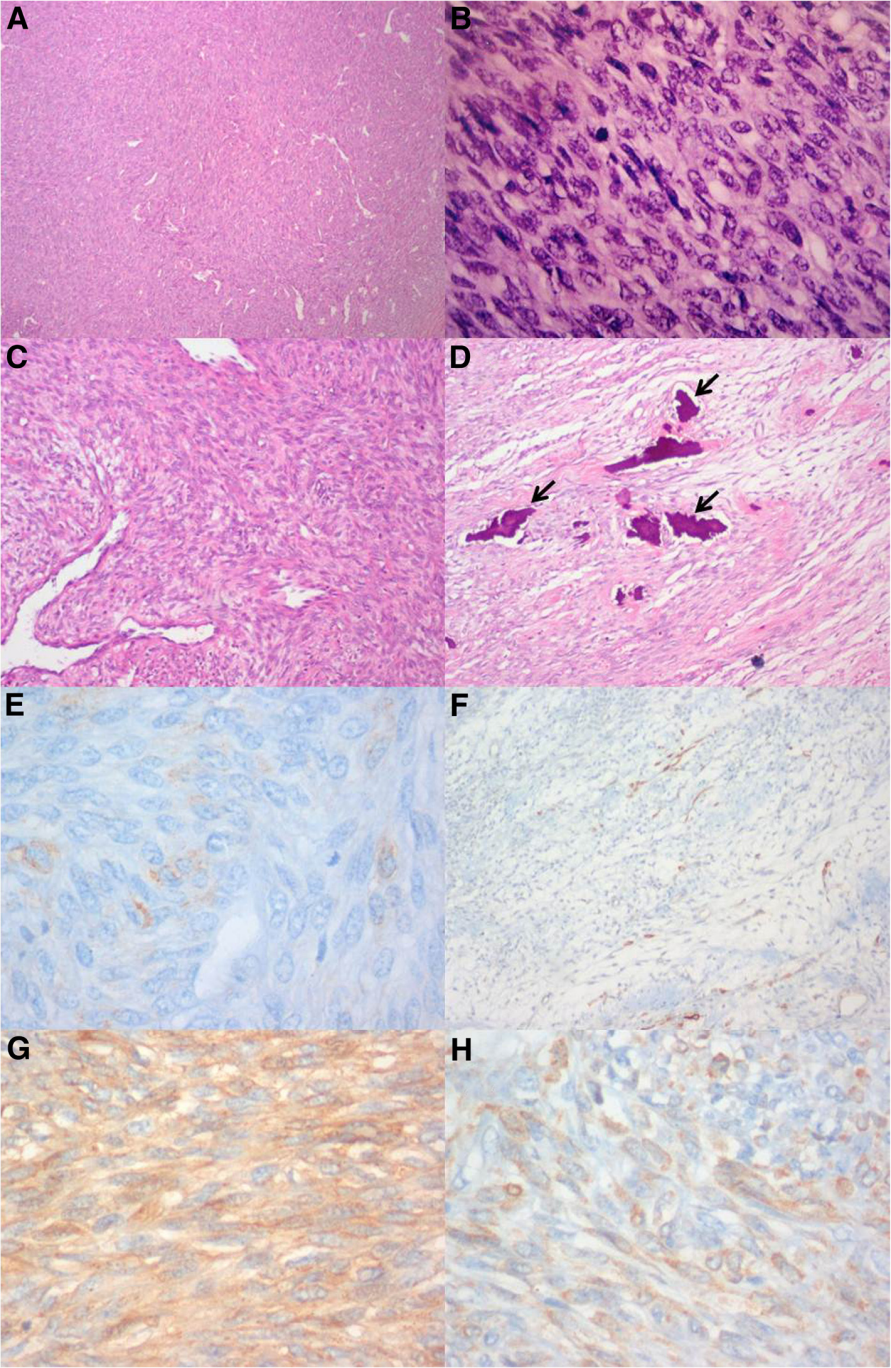
**Histological appearance of the tumor. (A)** Photomicrograph of a low-power magnification (40×) of a hematoxylin and eosin-stained section showing numerous highly packed cells forming densely cellular sheets and vague fascicles. **(B)** High-power magnification (400×) photomicrograph showing a hematoxylin and eosin-stained section with spindle cells with oval nuclei and scarce cytoplasm. These cells are uniform and relatively small, characterizing the monophasic “fibrous variant” of synovial sarcoma. **(C)** High-power magnification (400×) photomicrograph of a hematoxylin and eosin-stained section showing focal areas of a prominent hemangiopericytomatous vascular pattern, which is a frequent finding in synovial sarcoma. **(D)** Intermediate-power magnification (100×) photomicrograph of a hematoxylin and eosin-stained section showing focal tumor calcifications (arrows), which is also a relatively frequent finding in these tumors. **(E)** High-power magnification (400×) photomicrograph of an immunohistochemical section marking epithelial membrane antigen outlining the surface of the sarcomatous cells, which is typical of synovial sarcoma. **(F)** Intermediate-power magnification (100×) photomicrograph of an immunohistochemical section marking cytokeratin 7 (CK7) showing CK7-positive cells either isolated or in cords. **(G)** High-power magnification (400×) photomicrograph of an immunohistochemical section marking CD99, which is staining the surfaces of tumor spindle cells. **(H)** High-power magnification (400×) photomicrograph of an immunohistochemical section marking B-cell lymphoma 2 (BCL-2), showing diffuse staining of the tumor.

Subsequently, with the patient under general anesthesia, the mass was excised via a palmar approach, preserving the flexor apparatus of the fingers as well as all major vessels and nerves (Figures 6 and 7). Macroscopically, the tumor was grayish and had a maximum length of approximately 10cm (Figure 6). The tumor was multinodular and rubbery and seemed to be circumscribed by a fibrous pseudocapsule. The post-operative period was uneventful. Recovery was fast, and the patient had no significant functional or aesthetic impairment (Figure 8). She declined any physiotherapy treatments. Her only complaint was pain over the surgical scar that subsided approximately 6 months post-operatively. Pathological examination revealed the typical appearance of synovial sarcoma of the monophasic type (Figure 9). The specimen margins were found free of tumor cells. There was no evidence of systemic disease on the computed tomography (CT) scan of the thorax, abdomen and pelvis that had been done pre-operatively. The patient underwent post-operative radiotherapy and chemotherapy with good tolerance. Adjuvant chemotherapy consisted of one cycle of ifosfamide and doxorubicin following the recommendations described by Kampe *et al*. [6]. Ifosfamide was given for 6 days at a dose of 14g/m^2^. An equimolar dose of mesna was combined with the first dose of ifosfamide of 2g/m^2^. These two drugs were given as a 4-hour intravenous bolus. The subsequent ifosfamide treatment was given at the rate of 2g/m^2^/24 hours by continuous intravenous infusion. The patient received an additional dose of mesna at the end of the sixth day. Doxorubicin was administered as a 48-hour continuous intravenous infusion, corresponding to a total dose of 60mg/m^2^. Adjuvant radiotherapy was delivered at the end of chemotherapy, including not only the tumor excision site but also a 3cm margin. A total dose of 60 Gy was applied, fractioned in 2.0 Gy daily, 5 days per week, during 6 weeks.

**Figure 10:**
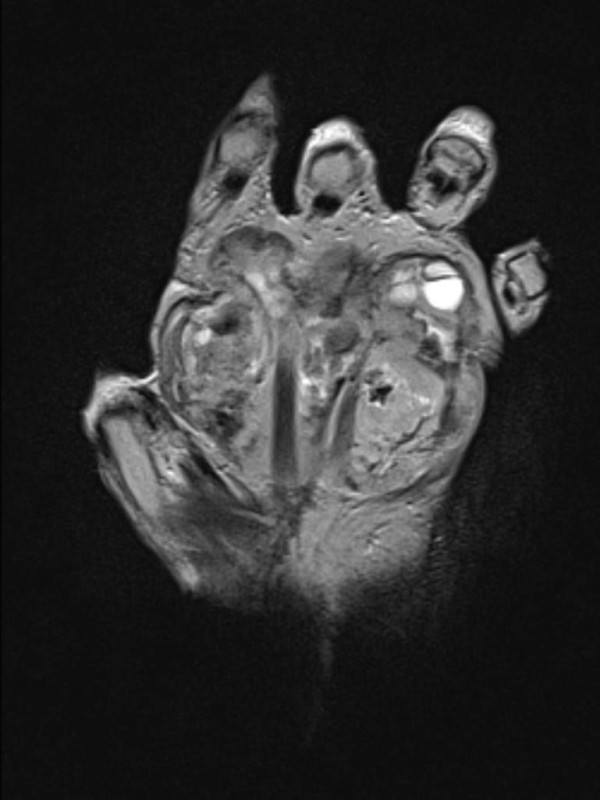
Magnetic resonance imaging scan of the hand showing tumor recurrence with a large hypervascular mass and foci of necrosis that occupied most of the anterior compartments of the hand and encroached into the metacarpal and carpal bones.

The patient was followed at the hand clinic at regular intervals for 9 years. The CT scans of the thorax, abdomen and pelvis that were done regularly during this period failed to show any evidence of systemic disease. She then decided to stop coming to the outpatient clinic, arguing that she saw no point in going as she felt perfectly well. Eleven and one-half years after surgery, the patient returned to the outpatient clinic with a recurrence of a sarcomatous mass in her left hand. MRI of the hand showed a large hypervascular mass with foci of necrosis that occupied most of the anterior compartments of the hand and encroached into the metacarpal and carpal bones (Figure 10). No evidence of metastasis was found.

A multidisciplinary team decided to propose left-hand amputation, which the patient declined. Twelve years after the initial diagnosis, she decided to stop coming to the outpatient clinic again and rebuffed any further contact. Six months later, she was found dead at her home, having had a massive hemorrhage from the recurrence in her hand.

## Discussion

Synovial sarcomas are malignant, high-grade, soft-tissue neoplasms that are estimated to represent between 5% and 10% of all soft-tissue sarcomas [3,7]. The estimated incidence of this tumor in the general population is 2.75 per 100,000 [3]. In fact, in adults, synovial sarcoma is the fourth most common type of sarcoma after malignant fibrous histiocytoma, liposarcoma and rhabdomyosarcoma [3]. In the United States alone, approximately 800 new cases are diagnosed each year [3,7]. In children, synovial sarcoma incidence is second only to rhabdomyosarcoma in terms of soft-tissue sarcoma [8]. Approximately one-third of synovial sarcomas occur in the first two decades of life [3,7,8]. This tumor is most prevalent in adolescents and adults between 15 and 40 years of age [3,7,8]. Concerning gender incidence, the male:female ratio is 1.2:1, with males being more frequently affected. Synovial sarcoma has a similar incidence in all ethnic groups [2,3,7,8].

This malignancy is usually located close to the large joints of the extremities, especially the lower extremities and in particular around the knee and ankle [2,3,7,8]. Other joints that are commonly affected are the shoulder and the hip. Most often it arises in the para-articular regions, usually in close association with tendon sheaths, bursae and joint capsules [2,3,7,8]. However, it seldom involves the joints themselves [3]. In addition, contrary to what its name might suggest, synovial sarcoma also occurs in areas with no apparent relation to synovial structures, such as the heart, pericardium, pleura, lung, mediastinum, larynx, peritoneal cavity and abdominal wall [3]. In the hand, this tumor is more frequently found in the carpal region than in the fingers [2,3,7,8].

According to most authors, delay in diagnosis is very frequent [3]. In the majority of cases, the presence of a clinically detectable tumor prior to surgery is estimated to range from 2 to 4 years, but an insidiously growing mass or pain at the tumor site has been noted for as long as 20 years prior to initiation of proper treatment [3,4]. Recently, it has been shown that the occurrence of long-standing pain at the tumor site preceding the development of a bulge is significantly more common with synovial sarcomas than with other sarcomas [9]. The imaging appearance is nonspecific, and in all cases a biopsy is necessary to confirm the diagnosis [3,10,11].

Histologically, synovial sarcoma is typically characterized by epithelium-like and/or spindle cell components arranged in a biphasic or monophasic pattern, although a poorly differentiated variant has also been described recently [12]. The biphasic pattern is considered the “classic” type and is generally recognizable by the coexistence of morphologically different but genetically similar epithelial cells and fibroblast-like spindle cells [3]. The monophasic type is closely related to the biphasic type and represents merely one extreme of its morphological spectrum, sharing phenotypical features identical to the spindle-cell portion or the epithelium-like component, corresponding to the monophasic fibrous variant or to the monophasic epithelial variant, respectively [3]. Histologically, the poorly differentiated type is composed mostly of small, solidly packed, oval or spindle-shaped cells that seem to have an intermediate phenotype between epithelial and spindle cells, often with scant differentiation, simulating other neoplasms, namely, angiosarcoma or small-cell carcinoma [3].

Immunohistochemically, the majority of synovial sarcomas express cytokeratins, epithelial membrane antigen, calponin, B-cell lymphoma 2 (BCL-2) and CD-99. Vimentin can also be found in the spindle cells of these tumors. These markers can help differentiate synovial sarcomas from other sarcomas [12,13].

Although microscopic resemblance to the developing synovium was initially suggested, its origin from preformed synovial tissues remains to be proven [12,13]. Owing to the similarity between synovial sarcoma tumor cells and primitive synoviocytes, the term *synovial sarcoma* was coined [12,13]. However, most of these tumors occur outside the joints themselves and bear no resemblance to synovial structures either ultrastructurally or immunohistochemically [12]. It has been proposed that synovial sarcoma arises from the pluripotential mesenchyme of the limb bud [3].

A particular chromosomal translocation t(X;18) has been noted in over 90% of cases, both in adults and in children [12,14]. Although synovial sarcomas can be graded histologically according to mitotic index, percentage of necrosis and tumor differentiation, almost all authors believe these tumors should always be regarded as high-grade sarcomas [12,14].

Synovial sarcomas not only are locally aggressive but also have a higher metastatic potential than most other soft-tissue sarcomas. Hence, the overall prognosis for synovial sarcoma patients is poor [2,8,12]. In fact, according to most reports, notwithstanding intensive multimodal therapy, including surgery, chemotherapy and radiotherapy, the outcomes of these patients have changed little in the past two decades [3,15]. According to the literature, local recurrence and/or metastatic disease are found in nearly 80% of patients [2,8,12,15]. Several factors have been associated with a higher recurrence risk. These factors include older age, larger tumor size (> 5cm), truncal location or proximal tumors in the limbs, male sex, bone or neurovascular invasion, incomplete excision on pathological examination, p53 overexpression, high proliferative index and, more recently, specific SYT-SSX fusion types [16,17]. Our patient presented with only two of these risk factors, which may help explain the unusual long survival time observed. The most common site for metastasis is the lung [4,18]. Lymph node involvement has been reported to occur in as many as 27% of patients [18].

Surgical resection is the definitive choice of treatment for primary synovial sarcomas and has been shown to both control local recurrence and prevent systemic dissemination [2,8,12]. Unfortunately, the minimal acceptable margin has not been clearly established, and the surgeon must be aware of the possibility of microscopic infiltration of tumor cells into the pseudocapsule of the tumor [1,3,10]. Many investigators have suggested 1 cm to 2cm resection margins [1-3,10]. Because of proximity to the joints, the ablation can consist of either tenosynovectomy and/or post-operative radiotherapy and/or chemotherapy, or simply extremity segment amputation [3]. If ablation and tenosynovectomy are selected to retain maximal function, there might be a compromise of soft-tissue cover over tendons and neurovascular pedicles [3]. When proximity to critical anatomical structures and patient desire do not allow the surgeon to obtain adequate surgical margins, isolated limb perfusion and radiotherapy must be considered, as they can potentially prevent amputation. In all cases, multidisciplinary discussion of adjuvant therapies that may prevent amputation must occur prior to surgery [1-3,10,18]. Flaps are also an important option to bear in mind, as they can provide coverage of vital anatomical structures, as well as minimize the effects of radiation injury on these structures [19]. Discussion of the most adequate curative procedure, knowledge of the available reconstructive options, consideration of possible comorbidities and patient wishes for limb preservation must all be taken into account before surgery [19].

The efficacy of adjuvant chemotherapy is still a matter of intense debate [18]. Similarly, radiotherapy is associated with a higher rate of local disease control, but not with better survival rates [3,18].

The presence of metastasis is considered the major cause of poor outcome, and several reports describing the results of current therapy showed a 5-year survival rate of around 27% to 55% [2,8,12]. Factors determining a worse prognosis include tumor diameter > 5cm, inadequate surgical resection, local recurrence, patient age over 20 years, monophasic variant and high mitotic activity [2,5,8,15]. Therefore, such a prolonged longevity as we observed in our patient is unfortunately not the most common result [3].

## Conclusion

Awareness of this rare tumor by anyone dealing with hand pathology can hasten diagnosis, and this, in turn, can potentially increase survival [9]. Therefore, a high index of suspicion for this disease should be kept in mind, particularly when evaluating young people, as this is the most commonly affected group [3,7-9].

## Consent

Written informed consent was obtained from the patient’s next-of-kin for publication of this case report and any accompanying images. A copy of the written consent is available for review by the Editor-in-Chief of this journal.

## Competing interests

The authors declare that they have no competing interests.

## Authors’ contributions

DC played a major role in writing the manuscript and analyzed the patient data. AIR and MM played major roles in analyzing the patient data and reviewing the manuscript. CA aided in the editing of the manuscript and analyzed the patient data. CM followed up the patient, aided in the editing of the manuscript and analyzed the patient data. MM Mendes followed up the patient, edited the manuscript and analyzed the patient data. MM Mouzinho operated on the patient, participated in follow-up, played a major role in writing the manuscript and analyzed the patient data. All authors read and approved the final manuscript.
